# Evaluation of Kinesiophobia and Its Correlations with Pain and Fatigue in Joint Hypermobility Syndrome/Ehlers-Danlos Syndrome Hypermobility Type

**DOI:** 10.1155/2013/580460

**Published:** 2013-07-14

**Authors:** Claudia Celletti, Marco Castori, Giuseppe La Torre, Filippo Camerota

**Affiliations:** ^1^Physical Medicine and Rehabilitation, Department of Orthopaedics, Sapienza University, Umberto I Hospital, Piazzale Aldo Moro 3, 00185 Rome, Italy; ^2^Medical Genetics, Department of Molecular Medicine, Sapienza University, San Camillo-Forlanini Hospital, 00151 Rome, Italy; ^3^Department of Public Health and Infectious Diseases, Sapienza University, Umberto I Hospital, 00185 Rome, Italy

## Abstract

Ehlers-Danlos syndrome hypermobility type a. k. a. joint hypermobility syndrome (JHS/EDS-HT) is a hereditary musculoskeletal disorder associating generalized joint hypermobility with chronic pain. Anecdotal reports suggest a prominent role for *kinesiophobia* in disease manifestations, but no study has systematically addressed this point. *Objective*. To investigate the impact of *kinesiophobia* and its relationship with pain, fatigue, and quality of life in JHS/EDS-HT. *Design*. Cross-sectional study. *Subjects/Patients*. 42 patients (40 female and 2 male) with JHS/EDS-HT diagnosis following standardized diagnostic criteria were selected. *Methods*. Disease features were analyzed by means of specific questionnaires and scales evaluating kinesiophobia, pain, fatigue, and quality of life. The relationships among variables were investigated using the Spearman bivariate analysis. *Results*. *Kinesiophobia* resulted predominantly in the patients' sample. The values of *kinesiophobia* did not correlate with intensity of pain, quality of life, and (or) the single component of fatigue. A strong correlation was discovered between *kinesiophobia* and general severity of fatigue. *Conclusions*. In JHS/EDS-HT, the onset of pain-avoiding strategies is related to the presence of pain but not to its intensity. The clear-cut correlation between *kinesiophobia* and severity of fatigue suggests a direct link between musculoskeletal pain and fatigue. In JHS/EDS-HT, the underlying mechanism is likely to be facilitated by primary disease characteristics, including hypotonia.

## 1. Introduction

 Ehlers-Danlos syndrome (EDS) is an umbrella term for various hereditary connective tissue disorders (HCTDs) mainly characterized by congenital joint hypermobility, skin hyperextensibility, and tissue fragility. Among the six major forms [1], the classic and the hypermobility (EDS-HT) types are considered the most common. EDS-HT typically features joint laxity and related complications, chronic/recurrent limb pain, and minor skin involvement [1], although its extended clinical spectrum covers a wide variety of functional somatic syndromes [2]. An international panel of experts now considers EDS-HT one and the same as joint hypermobility syndrome (JHS) [3], and this overlap is particularly evident in adulthood. The diagnosis of JHS/EDS-HT still remains unsupported by molecular testing, with the exception of a very few cases purportedly mutated in *COL3A1* and *TNXB* [4–6]. More recently, the relevance of previous molecular findings has been further reduced by the separation of patients mutated in *TNXB*, who are now grouped under a distinct EDS subtype (i.e., *TNXB*-deficient EDS) [7]. At the moment, recognition of JHS/EDS-HI is uniquely based on clinical diagnostic criteria [1, 8], which needs urgent revision [9].

Pain and fatigue are considered relevant determinants of disability in JHS/EDS-HT [10]. However, their manifestations and pathophysiology remain poorly characterized. Accumulated data demonstrate that pain is often chronic and widespread in EDS [11] and associates positively with residual joint hypermobility, dislocations, previous surgery, and low nocturnal sleep quality [12]. Limb pain, though considered originating mostly from primary joint damage, shows some neuropathic features in ~2/3 patients [13]. Such preliminary findings anticipate a complex pathophysiology for pain in JHS/EDS-HT, in which cognitive aspects are likely to strongly affect quality of life. Accordingly, Rombaut et al. [14] commonly encountered fear of falling among women with JHS/EDS-HT. 

Chronic musculoskeletal conditions, predominantly characterized by chronic pain, are often associated with fear [15]. Fear is the emotional reaction to a specific, identifiable, and immediate threat, such as a dangerous animal or injury [16]. Pain-related fear can be defined as the fear that emerges when stimuli that are related to pain are perceived as a main threat [16]. Fear in relation to pain is described in three constructs: pain-related fear, fear of movement, and kinesiophobia [17]. Kinesiophobia is the most extreme form of fear of movement, and is defined as an excessive, irrational and debilitating fear of physical movement and activity resulting from a feeling of vulnerability to painful injury or reinjury, and it has been reported as a common feature of patients with CFS [18], fibromyalgia, and chronic low back pain [19]. Fear-avoidance is said to play a role in the so-called deconditioning syndrome which can either be expressed in a weakened muscle strength, or disordered muscle coordination, during physical activity [16].

In this study, we carried out a questionnaire on 42 JHS/EDS-HT patients in order to evaluate the presence and severity of *kinesiophobia* and to analyze its relationship with pain, fatigue, and quality of life (QoL). Implications of the results in the treatment of JHS/EDS-HT are discussed below. 

## 2. Patients and Methods

### 2.1. Patient Selection

All patients studied have attended a multidisciplinary service dedicated to HCTDs and were followed into the “joint hypermobility” outpatient clinic in the Division of Physical Medicine and Rehabilitation of the Umberto I University Hospital (Rome, Italy) and into the clinical genetics outpatient clinic at the Medical Genetics of the San Camillo-Forlanini Hospital (Rome, Italy). Diagnosis was based on published diagnostic criteria including the Brighton criteria for JHS [8] and the Villefranche criteria for EDS-HT [1]. Patients were included if they met at least one of these two sets. In our clinical practice, the Brighton criteria are the most stringent for young-adult, adult, and elderly patients, while the Villefranche criteria are the best for individuals in the pediatric age group. For this study, JHM was mainly assessed applying the Beighton score [20]. Further joint or group of joints were equally evaluated although, at the moment, their status do not influence diagnosis establishment. The Beighton score is a 9-point evaluation with attribution of one point in the presence of any of the following features: (a) passive apposition of the thumb to the flexor aspect of the forearm (one point for each hand), (b) passive dorsiflexion of the V finger beyond 90° (one point for each hand), (c) hyperextension of the elbow beyond 10° (one point for each arm), (d) hyperextension of the knee beyond 10° (one point for each leg), and (e) forward flexion of the trunk with the knees extended and the palms resting flat on the floor. Skin/superficial connective tissue features were assessed qualitatively on the basis of accumulated experience by palpation and gentle stretching of the skin at the volar aspect of the palm (at the IV metacarpal) and/or of the forearm. Other HCTDs were excluded clinically. Patients were also evaluated to search other secondary symptoms of the pathology that are showed in Table 1. Individuals with incomplete diagnosis were equally excluded. Thus, a group of patients with insufficient features of JHS for a firm clinical diagnosis based on the available diagnostic criteria, but likely to be liable to develop full-blown JHS, were not included in this study. Pregnant women and patients older than 60, or younger than 15, were not included in order to better homogenize the sample.

### 2.2. Evaluation Tools

In order to evaluate kinesiophobia, pain, and fatigue, all patients were asked to fill in a series of questionnaires including the Tampa Scale Italian version (TSK-I) [21], the Fatigue Severity Scale (FSS) [22], the Multidimensional Fatigue Inventory Scale (MFI-20) [23], and the Numeric Rating Scale (NRS-11) for pain [24]. Data were also compared with quality of life, which was evaluated by the Medical Outcome Study Short Form-36 (SF-36) [25].

#### 2.2.1. Kinesiophobia

TSK-I is the most widely used questionnaire to assess pain and pain-related fear of movement in subjects with musculoskeletal complaints [26, 27], and this has been translated into and validated in different languages including Italian [21]. TSK-I is divided into two sub-scales: evaluating *activity avoidance* (TSK-AA: a belief that activities causing pain should be avoided) and *harm* (TSK-H: a belief that pain is a sign of bodily damage), respectively. TSK-I is able to distinguish the fear of movement domain from other conceptual domains such as pain and functional alteration. The original version of the TSK-I questionnaire comprises 17 items to assess the subjective rating of kinesiophobia [28]. Each item has a four-point Likert scale with scoring alternatives ranging from “strongly disagree” to “strongly agree.” A total sum is calculated after inversion of the individual scores of items 4, 8, 12, and 16. The total score ranges between 17 and 68. A high TSK-I value indicates a high degree of kinesiophobia. In the Italian version [21], items 4, 8, 12, and 16 were excluded with a total score of 52 and a maximum score for *activity avoidance* and *harm* of 24 and 28, respectively. 

#### 2.2.2. Fatigue

FSS is a scale quantifying fatigue intensity, which has been used in different chronic conditions, such as multiple sclerosis and systemic lupus erythematosus [29], and shows high internal consistency and validity. FSS comprises 9 items with a 7-point response format that indicates the degree of agreement with each statement [22]. MFI-20 is a 20-item self-reporting instrument designed to measure fatigue [23]. It covers the following dimensions: general fatigue (GF), physical fatigue (PF), mental fatigue (MF), reduced motivation (RM), and reduced activity (RA). The questionnaire is constructed with an equal number of questions for each of the five suggested dimensions. It has been demonstrated that it is reliable and valid and has been tested in various conditions, such as cancer or chronic fatigued patients [23].

#### 2.2.3. Pain

NRS is a rapid-to-administrate 11-point numeric scale used to roughly measure any kind of pain with a score ranging from 0 (no pain) to 10 (acute pain).

#### 2.2.4. Quality of Life

The Medical Outcome Study 36-item Short-Form Health Survey (SF-36) is a multipurpose, short form health survey to evaluate aspects of health most closely related to quality of life with 36 questions that measure 8 conceptual domains: physical functioning, physical limitation, bodily pain, general health, vitality, social functioning, emotional limitation, and mental health. The raw scores in each domain are transformed into 0 to 100 scale with higher scores indicating better quality of life [25]. The questionnaire has been translated into Italian and thoroughly validated in the Italian context [29].

### 2.3. Statistical Analysis

Statistical analysis was conducted with the SPSS software package for Windows, version 19.0. The Kolmogorov-Smirnov probability test was used to assess the normality of the distributions. The FSS mean score was compared with normal healthy adult scores extracted from Krupp et al. [22] using the one sample *t*-test. SF-36 scores were compared with a sample of the normal Italian population [29] using the one sample *t*-test.

The Spearman bivariation analysis was conducted in order to test independent variables related to dependent ones. Variables assumed as independent included age, sex, Beighton scores, FSS mean scores, the four subscores of MFI-20, the NRS mean scores, and the SF-36 in all subforms, while TSK-I, TSK-AA, and TSK-H scores were considered *dependent *variables. Subsequently, a separate multivariate linear regression analysis was performed for TSK-I, TSK-AA, and TSK-H as dependent variables, using the Backward elimination stepwise method and including only independent variables that show *P* < 0.250 at the univariate analyses. The significance level was set at *P* < 0.05.

## 3. Results

**Figure 1:**
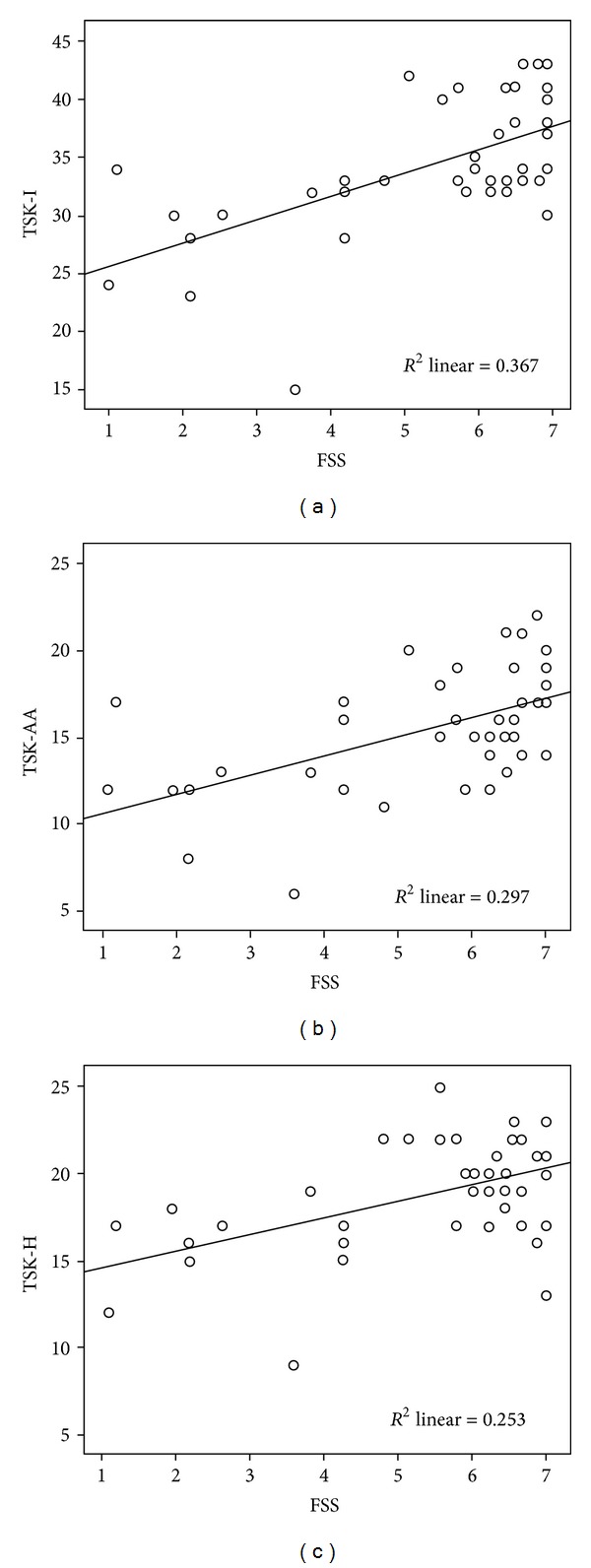
Correlation between Fatigue Severity Scale (FSS) and kinesiophobia as total score TSK-I (a) and in the activity avoidance TSK-AA (b) and in the harm TSK-H (c) subscales.

Forty-two patients (40 females and 2 males; mean age at evaluation: 32.80 ± 13.23 years) were selected. Clinical characteristics of the patient samples are summarized in Table 1.  Table 2 illustrates numerical values of the following variables used for further statistical analysis: Beighton score, TSK-I, FSS, MFI-20, NRS (pain), and SF-36. In the patient population, the mean score of TSK-I and its sub-scales (TSK-AA and TSK-H) were higher than the value previously fixed as the cut off for establishing the presence of fear of movement [15], with the 93% of patients with high score values. FSS and FS-36 and all its domains were compared with those previously registered in the general population [22, 29]. All values were higher in the patient samples with a significance of *P* < 0.001. Results of the Spearman bivariation analysis comparing the TSK-I, TSK-AA, and TSK-H values with the selected variables are summarized in Table 3.  Table 4 shows results of the multivariate linear regression analysis by the Backward elimination stepwise method. The most consistent association was the one between TSK-I (including both domains) and FSS (Figure 1). Association between TSK-I and some MFI-20 domains appeared weaker, though still statistically significant in relation to the Spearman bivariation analysis. 

## 4. Discussion

Pain-related fear is a particular characteristic of patients with musculoskeletal disorders [15] and plays an important role in explaining disability and in transition from acute to chronic musculoskeletal pain [30]. From this perspective, *kinesiophobia* (i.e., cognitive fear of movement or reinjury) can lead to the stopping/reduction of various activities thought to generate pain with progressive limitation of mobility in some individuals. The consequent disuse and deconditioning generate further loss of muscle tone, flexibility, and aerobic capacity, which may explain (bearing in mind the population under consideration) the transition to the third disease phase in JHS/EDS-HT [31, 32] symptom progression. In this phase, psychological and physical disability is marked with many patients suffering from anxiety, depression, and somatosensory amplification [33] and some obliged to use a wheelchair. 

Overall, the present study, conducted through a questionnaire-based investigation into 42 patients, in which there is a preponderance of females described as characteristic in JHS/EDS-HT even if the mechanism underlying is unknown [34], confirmed the hypothesis that *kinesiophobia* is a common symptom in JHS/EDS-HT. We also confirmed global deterioration of the QoL, moderate/severe bodily pain and marked fatigue in our patient cohort. Since pain and fatigue have previously been proposed as relevant factors determining disability in JHS/EDS-HT [35], we tried to compare the severity of *kinesiophobia* with the QoL, intensity of pain and fatigue. Although the bivariate analysis identified a series of possible correlations (Table 3), further refining by multivariate linear regression analysis (Table 4) confirmed correlation with only general severity of fatigue (i.e., FSS). Lack of correlation with intensity of pain suggests an intriguing relationship between *kinesiophobia* and pathophysiology of chronic pain in JHS/EDS-HT. In fact, one could expect that the impact of pain-avoiding strategies is directly linked to the intensity of perceived pain. This does not hold true in our sample, where the onset of *kinesiophobia* is influenced by the presence of pain, but not by its severity. Therefore, in JHS/EDS-HT, it is plausible that individual coping strategies are more relevant than the intensity and/or frequency of the pain stimulus in generating the psychological and physical disability related to pain-avoiding behaviours. 

Conversely, *kinesiophobia* strongly relates with severity of fatigue, but not its single components in our sample. Such a result suggests a direct link between adoption of pain-avoiding strategies and chronic fatigue. Accordingly, it could be hypothesized that *kinesiophobia* may contribute to the progression and, perhaps, onset of fatigue by bodily disuse secondary to decreased physical effort. Therefore, a three-phase model of pain-*kinesiophobia*-fatigue can be proposed by which, in predisposed individuals, repeated musculoskeletal traumatism exacerbated by joint hypermobility generates pain-avoiding strategies, which, in turn, cause/aggravate fatigue. This mechanism, which may be considered acting in many chronic pain conditions, appears more pronounced in JHS/EDS-HT, as hypotonia and hypermobility are primary features of the disease, and the effects of pain-avoiding strategies are likely to appear more rapidly and to be more severe. Recent finding that muscle weakness is associated with fatigue in EDS is in line with this assumption [35]. However, this model of pain-*kinesiophobia*-fatigue cannot explain the entire spectrum of clinical variability in JHS/EDS-HT. In fact, by tracing its natural history, the onset of fatigue could be independent of joint instability complications and limb pain, at least in some cases [36]. The reason why, in our sample, *kinesiophobia* does not correlate with QoL remains unexplained. This probably reflects that QoL tools, such as the SF-36, measure multidimensional variables that are not limited to disability only. Consequently, the effects that *kinesiophobia* has on them are too small to be statistically significant. 

This study demonstrates once more the urgent need for evaluating and treating JHS/EDS-HT patients within multidisciplinary teams comprising a variety of specialists (who can focus on pain and fatigue) including physiatrists, physical and occupational therapists, clinical psychologists, neuropsychologists, pain specialists, and rheumatologists. In fact, the classic approach of treating JHS/EDS-HT-related pain based on a combination of physical therapy and adjuvant pharmacologic support should be associated with and, hopefully, substituted, at least in terms of prevention, by regular physical exercise and cognitive therapies. Various studies have demonstrated that exercise and fitness are beneficial in a biomedical sense of maturation, strengthening, and healing of bones, tendons, and muscle, while deconditioning refers to a progressive process of worsening physical fitness as reduced muscular activity [15]. In JHS/EDS-HT, in particular, doing moderate and continuous physical activity seems to be useful in order to keep joint hypermobility and muscle tone, and to reduce pain, fatigue, and fear of movement.

An individualized, modified, and therapeutic programme involving a multidisciplinary team is recommended to prevent chronic pain and deconditioning and thereby reduce suffering in JHS/EDS-HT patients.

## Figures and Tables

**Table 1:** General characteristics.

Characteristic	Frequency (total = 42)	%
Gender (female/male)	40/2	95.2/4.8
Positive family history	19	45.2
Contortionism in pediatric age	35	83.3
Motor delay/clumsiness	9	21.4
Residual joint hypermobility (Beighton ≥4)	31	73.8
Recurrent (≥3) joint dislocations	31	73.8
Recurrent (≥3) soft tissue lesions	18	42.8
Chronic back pain	33	78.6
Chronic arthralgias	35	83.3
Chronic myalgias	36	85.7
Chronic fatigue	37	88.1
Recurrent headaches	32	76.2
Unrefreshing sleep	31	73.8
Impaired memory/concentration	24	57.1
Velvety/smooth skin	31	73.8
Hyperextensible skin	11	26.2
Easy bruising	29	69.0
Eyelid ptosis	17	40.5
Varicose veins/hemorrhoids	7	16.7
Hernias	2	4.8
Uterine/vesical/rectal prolapse	4	9.5

**Table 2:** Rough data of the Beighton score, TSK-I, MFI-20, FSS, NRS (pain), and FS-36.

Variable	Mean ± SD (range)
Beighton score	5.47 ± 1.97 (0–9)
TSK-I	34.40 ± 5.85 (13–52)
TSK-AA	15.52 ± 3.48 (6–24)
TSK-H	18.88 ± 3.26 (7–28)
MFI-20, GF	17.59 ± 3.12 (4–20)
MFI-20, PF	16.90 ± 2.86 (4–20)
MFI-20, RA	14.33 ± 4.04 (4–20)
MFI-20, RM	12.71 ± 2.54 (4–20)
MFI-20, MF	14.26 ± 4.64 (4–20)
FSS	5.42 ± 1.77 (0–7)
NRS (pain)	7.09 ± 1.49 (4–10)
SF-36, PF	45.23 ± 25.42 (0–100)
SF-36, RP	17.26 ± 26.18 (0–100)
SF-36, BP	25.91 ± 21.73 (0–100)
SF-36, GH	26.45 ± 16.30 (0–100)
SF-36, VT	30.11 ± 20.01 (0–100)
SF-36, SF	38.39 ± 25.66 (0–100)
SF-36, RE	43.88 ± 40.43 (0–100)
SF-36, MH	55.52 ± 23.77 (0–100)

BP: bodily pain; FSS: Fatigue Severity Scale; GF: general fatigue; GH: general health; MF: mental fatigue; MFI: Multidimensional Fatigue Inventory; MH: mental health; NRS: Numeric Rating Scale; PF (SF-36): physical functioning; PF (MFI-20): physical fatigue; RA: reduced activity; RE: role-emotional; RM: reduced motivation; RP: role-physical; SF: social functioning; SF-36: short form 36; TSK-AA: Tampa Scale activity avoidance; TSK-H: Tampa Scale harm; TSK-I: Tampa Scale total score; VT: vitality.

**Table 3:** Results of the Spearman bivariation analysis comparing TSK-I, TSK-AA, and TSK-H values (as dependent variables) with different variables.

Variable	TSK-I	TSK-AA	TSK-H
Age	−0.79 (*P* = 0.62)	−0.267 (*P* = 0.088)	0.146 (*P* = 0.356)
Beighton score	−0.66 (*P* = 0.67)	0.022 (*P* = 0.889)	−0.109 (*P* = 0.49)
NRS	0.013 (*P* = 0.93)	0.068 (*P* = 0.667)	0.005 (*P* = 0.973)
FSS	0.558 (**P** < 0.001)	0.573 (**P** < 0.001)	0.388 (**P** = 0.01)
MFI-20, GF	0.323 (**P** = 0.037)	0.342 (**P** = 0.02)	0.226 (*P* = 0.15)
MFI-20, PF	0.289 (*P* = 0.06)	0.335 (**P** = 0.03)	0.209 (*P* = 0.18)
MFI-20, RA	0.227 (*P* = 0.14)	0.382 (**P** = 0.01)	0.064 (*P* = 0.68)
MFI-20, RM	0.057 (*P* = 0.72)	−0.022 (*P* = 0.888)	0.183 (*P* = 0.24)
MFI-20, MF	0.438 (**P** = 0.004)	0.498 (**P** = 0.001)	0.250 (*P* = 0.11)
SF-36, PF	−0.187 (*P* = 0.23)	−0.191 (*P* = 0.22)	−0.130 (*P* = 0.41)
SF-36, RP	−0.170 (*P* = 0.28)	−0.182 (*P* = 0.25)	−0.111 (*P* = 0.48)
SF-36, BP	−0.103 (*P* = 0.51)	−0.700 (*P* = 0.66)	−0.109 (*P* = 0.49)
SF-36, GH	−0.47 (*P* = 0.76)	−0.160 (*P* = 0.31)	0.087 (*P* = 0.58)
SF-36, VT	−0.323 (**P** = 0.03)	−0.293 (*P* = 0.06)	−0.266 (*P* = 0.08)
SF-36, SF	−0.222 (*P* = 0.15)	−0.213 (*P* = 0.17)	−0.169 (*P* = 0.28)
SF-36, RE	−0.109 (*P* = 0.49)	−0.202 (*P* = 0.20)	0.02 (*P* = 0.89)
SF-36, MH	−0.379 (**P** = 0.01)	−0.351 (**P** = 0.02)	−0.303 (*P* = 0.05)

Significant *P* values are in bold.

BP: bodily pain; FSS: fatigue severity scale; GF: general fatigue; GH: general health; MF: mental fatigue; MFI: multimensional fatigue inventory; MH: mental health; NRS: numeric rating scale; PF (SF-36): physical functioning; PF (MFI-20): physical fatigue; RA: reduced activity; RE: role-emotional; RM: reduced motivation; RP: role-physical; SF: social functioning; SF-36: short form 36; TSK-AA: Tampa Scale activity avoidance; TSK-H: Tampa Scale harm; TSK-I: Tampa Scale total score; VT: vitality.

**Table 4:** Multivariate linear regression analysis using the Backward elimination stepwise method.

Variable	TSK-I	TSK-AA	TSK-H
Age	−0.43 (*P* = 0.43)	−0.06 (*P* = 0.08)	0.023 (*P* = 0.499)
Sex	0.734 (*P* = 0.825)	0.986 (*P* = 0.592)	−0.498 (*P* = 0.789)
FSS	1.999 (**P** < 0.01)	1.075 (**P** = 0.000)	0.927 (**P** = 0.001)
MFI-20, GF	−0.101 (*P* = 0.81)	−0.090 (*P* = 0.670)	−0.045 (*P* = 0.858)
MFI-20, PF	0.15 (*P* = 0.679)	0.09 (*P* = 0.685)	0.03 (*P* = 0.90)
MFI-20, RA	−0.05 (*P* = 0.819)	0.157 (*P* = 0.231)	−0.184 (*P* = 0.159)
MFI-20, MF	0.05 (*P* = 0.84)	0.087 (*P* = 0.550)	−0.034 (*P* = 0.820)
SF-36, MH	−0.043 (*P* = 0.20)	−0.20 (*P* = 0.32)	−0.021 (*P* = 0.30)
SF-36, VT	−0.007 (*P* = 0.89)	0.007 (*P* = 0.085)	−0.018 (*P* = 0.53)
*R* ^²^ of the model	0.637	0.368	0.253

Significant *P* values are in bold.

FSS: fatigue severity scale; GF: general fatigue; MF: mental fatigue; MFI: multimensional fatigue inventory; MH: mental health; PF: physical fatigue; RA: reduced activity; SF-36: short form 36; TSK-AA: Tampa Scale activity avoidance; TSK-H: Tampa Scale harm; TSK-I: Tampa Scale total score; VT: vitality.
